# Powering the Immune System: Mitochondria in Immune Function and Deficiency

**DOI:** 10.1155/2014/164309

**Published:** 2014-09-21

**Authors:** Melissa A. Walker, Stefano Volpi, Katherine B. Sims, Jolan E. Walter, Elisabetta Traggiai

**Affiliations:** ^1^Department of Neurology, Massachusetts General Hospital, Boston, MA 02114, USA; ^2^Department of Neuroscience, Rehabilitation, Ophthalmology, Genetics, Maternal and Child Health (DINOGMI), University of Genoa, Genoa, 16147 Genova, Italy; ^3^Division of Allergy and Immunology, Department of Pediatrics, Massachusetts General Hospital for Children, Boston, MA 02114, USA; ^4^Novartis Institute for Research in Biomedicine, Basel, Switzerland

## Abstract

Mitochondria are critical subcellular organelles that are required for several metabolic processes, including oxidative phosphorylation, as well as signaling and tissue-specific processes. Current understanding of the role of mitochondria in both the innate and adaptive immune systems is expanding. Concurrently, immunodeficiencies arising from perturbation of mitochondrial elements are increasingly recognized. Recent observations of immune dysfunction and increased incidence of infection in patients with primary mitochondrial disorders further support an important role for mitochondria in the proper function of the immune system. Here we review current findings.

## 1. Introduction

Mitochondria represent a significant source of energy for eukaryotic cells. These organelles are present in all cell types and organ systems but are heterogenous in number, morphology [1], and molecular composition [2] at the tissue and even subcellular levels [3]. This raises the possibility of tissue- or system-specific roles for mitochondria and organ-specific pathobiology and clinical dysfunction.

Current understanding of the breadth of the cellular roles performed by mitochondria is expanding. Well-described mitochondrial cellular functions include aerobic metabolism and redox reactions via the electron transport chain (ETC), reactive oxygen species (ROS) homeostasis, mitochondrial uncoupling for heat production, and regulation of apoptosis and Ca++ homeostasis, as well as certain enzymatic functions such as heme [4] and steroid synthesis [5] and regulation of cellular metabolism. The diversity and scope of the human ETC (i.e., the known redundancy of core subunits in the ETC of mammals compared to bacteria and significant sequence homology noted between mammalian ETC subunits and known enzymes of divergent function) suggest that the human respiratory chain may execute multiple yet undescribed cellular or subcellular processes [6], which are cell or tissue specific. These cellular processes and mitochondrial heterogeneity are the product of mitofusin and mitophagy regulated by mitochondrial proteins coded by the nuclear DNA (over 1100 genes are predicted) [7, 8] and by the maternally inherited mitochondrial genome (22 transfer ribonucleic aids (tRNAs), 2 ribosomal ribonucleic acids (rRNAs), and 13 proteins) [9].

It is not surprising that—beyond their classical role in energy and metabolic mechanisms—recent data have proposed links between immune function and mitochondrial processes. Indeed, as described in this review, robust literature now exists implicating mitochondria in the proper function of the innate and adaptive immune system (as reviewed in [10–14]). Furthermore, monogenetic disorders of mitochondrial components may manifest with immune dysfunction. Conversely, immune deficiency and/or immune dysfunction are increasingly noted in individuals with primary mitochondrial disorders in which immunophenotypes have not traditionally been described.

## 2. Mitochondria and the Innate Immune System

A growing body of evidence links mitochondria to immunity in the basic scientific literature. Mitochondria function as signaling platforms and participate in effector responses [10], most notably in the innate immune response to cellular damage, stress, and infection by pathogens—particularly viral—via linkages to effectors of pattern recognition receptor (PRR) signaling [11]. PRRs recognize extracellular or intracellular highly conserved motifs termed pathogen-associated molecular patterns (PAMPs) presented by infectious agents. This recognition activates a signal cascade that ultimately results in an inflammatory response involving cytokines and other downstream effectors. Several PRRs are known to be direct targets of pathogens, resulting in the interference of the innate immune response to infection [15, 16]. PRRs include retinoic acid inducible gene- (RIG-I-) like receptors (RLRs), C-type lectin receptors (CLRs), Toll-like receptors (TLRs), and the nuclear oligomerization domain- (NOD-) like receptors (NLRs). Several downstream effectors of RLRs, NLRs, and TLRs link to the mitochondrion [12]. RLRs have been most strongly linked to viral immunity via interactions with the mitochondrial antiviral signaling protein (MAVS) that in turn activates NF-kappaB and IRF3 [17, 18]. NOD-like receptor family member X1 (NLRX1), a molecule that contains a mitochondrial addressing sequence [19, 20], has been shown to augment the MAVS antiviral response and localize to the mitochondrial matrix in biochemical assays [21, 22].

PRRs also recognize motifs of endogenous molecules released as a result of tissue injury in the absence of infection, so-called damage-associated molecular patterns (DAMPs) including cytosolic ATP [23], uric acid [24], and double-stranded DNA [25] and a subset of proteins [26, 27]. It is interesting to note that mitochondrial formyl peptides and mtDNA-encoded proteins are also potent DAMPs [28].

Additionally, mitochondria influence antiviral signaling via the production of reactive oxygen species (ROS). Overexpression of NLRX1 has been shown to induce reactive oxygen species (ROS), potentially via an interaction with a component of ETC complex III [21]. NOD-like receptor family, pyrin domain containing 3 (NLRP3) translocates from the endoplasmic reticulum to the mitochondrion when activated and mitochondrion-derived ROS are required for activation of NLRP3 inflammasome [29]. Likewise, TNF receptor associated factor 6 (TRAF6), a Toll-like receptor 4 signaling pathway intermediate, binds mitochondrion-localizing protein ECSIT (evolutionarily conserved signaling intermediate in Toll-like pathways), enhancing bactericidal activity [30].

## 3. Mitochondria and Metabolic Regulation of the Adaptive Immune System 

Extensive studies in T lymphocytes have shown that metabolism is tightly regulated during the entire lifespan of T cells [31]. Upon encountering an antigen lymphocytes undergo a process of activation, proliferation, and differentiation toward specific effector states. All these phenomena require changes in metabolism to enable cells to switch between a catabolic condition, such as the quiescent state of a memory cell, to the anabolic condition such as that required for activated cell division upon activation. Those metabolic changes are required not only for lymphocyte plasticity but also to influence T cell fate: modulation of cellular metabolism has been proven to promote T cell differentiation into specific lineages [32].

In the T cell, glucose is the prevalent source of energy for adenosine triphosphate (ATP) production. As a first step in the cytosol, glucose is converted into pyruvate and then subsequently used in the mitochondria in the tricarboxylic acid (TCA) cycle leading to ATP production through oxidative phosphorylation (OXPHOS) in the ETC. Alternatively to glucose, other sources of energy for T cell metablism/activation are glutamine or fatty acids [14, 31, 33].

Resting naïve T cells are metabolically quiescent and mainly rely on OXPHOS for energy production [13]. Upon cognate antigen encounter, while oxidative phosphorylation remains an effective source of energy, c-Myc upregulation [33] and estrogen related receptor alpa (EERalpha) signaling [34] activate the aerobic glycolytic pathway in order to meet the increased energetic needs (reviewed in [35]). Similarly to naïve T cells, memory CD8 T cells are relatively quiescent; however when antigen is reencountered they respond in faster and stronger fashion. This property has crucial consequence in the metabolic status of memory T cells. Indeed, several findings suggest that fatty acid oxidation and oxidative metabolism are crucial for memory generation, whereas glycolysis and glutaminolysis are required for effector T cell function [36, 37]. Memory CD8 T cells are characterized by the use of fatty acid oxidation as an alternative source of energy upon activation [37]: they have been shown to up-regulate carnitine palmitoyl transferase 1A (CPT1A), a mitochondrial membrane protein which controls beta-oxidation by regulating fatty acid transport across the outer mitochondrial membrane [37]. Moreover, mouse CD8 memory T cells have more mitochondrial mass than CD8 naïve T cells, which promotes oxidative as well as glycolytic capacity, allowing CD8 T memory cells to respond more rapidly upon secondary exposure to the antigen [38]. It has also been shown in human that CD8 effector memory T cells (CD8 EM) exhibit more glyceraldehyde-3-phosphate dehydrogenase (GAPDH) activity at early time points. Indeed, after CD28 engagement CD8 EM T cells rapidly produce IFN*γ* and concomitantly switch to glycolysis, being able to mount rapid secondary responses [39]. Additional evidence supports the role of glycolysis in the generation of terminally differentiated effector memory T cells, while glycolysis inhibition preserves the formation of long-lived memory CD8 T cells. These results reveal a new potential mechanism for improving the efficacy of cell-based therapy in cancer as well as chronic infectious diseases, such as hepatitis B [40]. Recently it has also been shown that in CD8 memory T cells the fatty acids (FA) needed for enhanced mitochondrial fatty acid oxidation (FAO) are not derived from external sources but rather from the intrinsic expression of the lysosomal hydrolase LAL which allows FA mobilization in CD8 memory recall responses [41].

Similar to what was observed in the resting and activated state, a different T cell metabolic signature has been observed in T effector populations. CD4 helper Th1, Th2, and Th17 specifically rely on glycolysis rather than mitochondrial metabolism, while T helper regulatory cells (Treg) show a parallel requirement for lipid metabolism, glycolysis, and OXPHOS [42–44]. In particular, the balance between Th17 and Treg differentiation appears to be regulated by the cell specific metabolic pathway through hypoxia inducible factor 1*α* (HIF1*α*) expression [35, 36]. Furthermore, the metabolic competence of T lymphocytes is reprogrammed in chronic inflammatory disorders. As an example, naïve CD4 T cells from rheumatoid arthritis patients are in a state of energy deprivation, generating less ATP and being more susceptible to apoptosis [45].

B lymphocyte metabolism has been poorly studied so far, but there is preliminary evidence that similar metabolic signatures might reflect their activation and differentiation states, even if in a different fashion compared to T lymphocytes. In fact, unlike in T cells, naïve resting mouse B cells show activation of glycolysis. Upon stimulation, via either TLR4 or B cell receptor (BCR), B cells upregulate both aerobic glycolysis and mitochondrial oxygen consumption (a signal of increased OXPHOS), through a c-Myc dependent pathway [46]. Inhibition of the entire glycolytic pathway, or selectively of mitochondrial oxidation, inhibits antibody production in vivo [47].

Recently Caro-Maldonado and colleagues have shown that mouse B cells are metabolically distinct from T lymphocytes in their response upon activation. Instead of shifting from oxidative phosphorylation to glycolysis, they increase their metabolism, maintaining a balance between the two pathways. Additionally, the same group demonstrated that anergic B cells are metabolically quiescent, while B cells chronically exposed to high BAFF level undergo a metabolic switch, rapidly increasing glycolysis on stimulation [48]. As an additional example, upon LPS stimulation, mouse B lymphocytes acquire extracellular glucose to support de novo lipogenesis necessary for proliferation and differentiation into plasma cells [49]. Finally, it has been demonstrated that autophagy, a self-digestion strategy that sustains cellular metabolism in a nutrient-depleted milieu, is a fundamental mechanism for plasma cell differentiation and function [50]. Despite these initial reports of metabolic pathways that influence mouse B cell differentiation including antibody class switch recombination, affinity maturation, memory generation, and plasma cell formation, metabolic dependence of human B cell pathways remains poorly understood.

**Figure 1:**
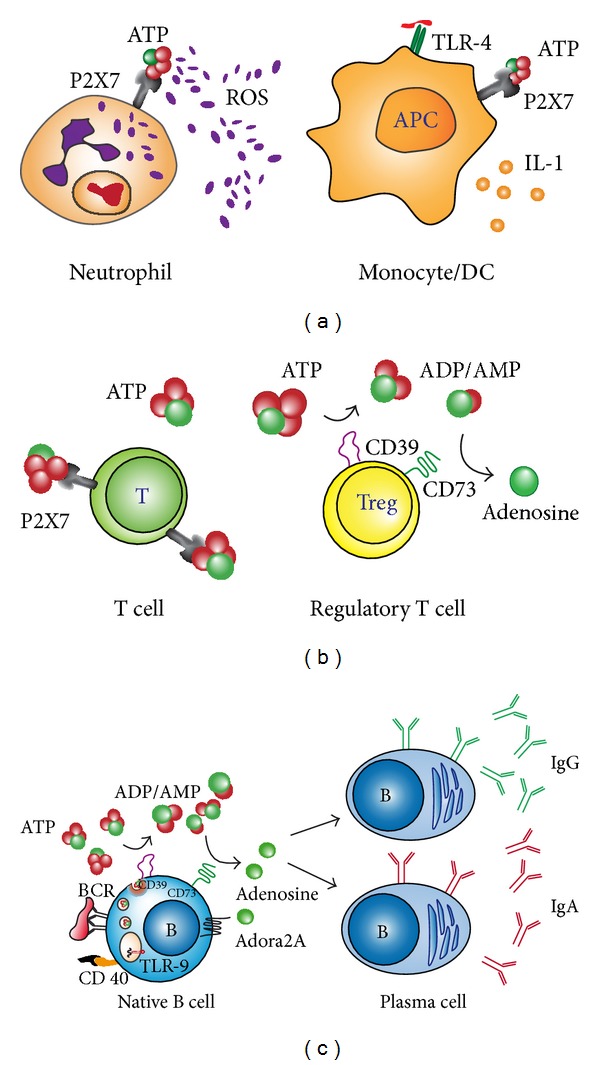
Immune cells regulation by extracellular purines. Nucleotides such as ATP and ADP are released from apoptotic cells or activated inflammatory cells through pannexin and connexin hemichannels or vesicular exocytosis. In the extracellular milieu ATP has a direct effect on both innate and adaptive immune cells through the activation of P2X and P2Y receptors, or after enzymatic conversion to adenosine, through its binding to adenosine receptor (ADORA). Examples of those functions are represented by granulocyte activation and IL8 secretion following P2Y receptors activation by ATP and IL1 secretion by monocyte and dendritic cells following concomitant P2X7 activation by ATP and TLR4 binding of bacterial LPS (a). Extracellular ATP promotes T lymphocytes activation by binding P2X7 receptors, a pathway modulated by regulatory T cell through ATP degradation to adenosine by the ectoenzymes expressed by those cells (b). In B lymphocyte adenosine production by the ectoenzymes expressed by subsets of B cells promotes class switch recombination and differentiation into IgG and IgA immunoglobulin secreting cells (c). (Modified from Schena et al. [59].)

Release of ATP in the extracellular milieu represents an important correlation between mitochondrial energy generation and lymphocyte function (Figure 1). Several groups have shown that after T cell activation via TCR, increased oxidative synthesis of ATP is followed by its release through pannexin 1 channels [51–54]. ATP in the extracellular space is rapidly hydrolyzed to ADP, AMP, and adenosine by ectoenzymes called ectonucleotidases [55]. These purine molecules represent the ligands for a class of membrane receptors called purinergic receptors that include ligand-gated ion channels specific for ATP (P2X receptors); G protein coupled receptors specific for ATP, UTP, and UDP-glucose (P2Y receptors); and G protein coupled receptor specific for adenosine (P1 receptors). The effect of secreted extracellular ATP and purinergic signaling contributes to the regulation of many cell types [56] acting mainly as a danger signal in the case of NLRP3 activation. IL1-*β* secretion by innate immune cell is regulated by ATP mediated P2X7 signaling and acts as a chemotactic molecule for neutrophil and macrophages migration [57]. Autocrine purinergic stimulation by ATP, through P2X receptors, also plays a crucial role in amplification of T cell signal amplification for proper cell activation and effector differentiation, acting through inhibition of Treg stability and favoring conversion towards TH17 cells [58].

In B lymphocytes, we have shown a similar autocrine regulation of cell effector function through purinergic signaling [59]. In human and mouse B cells, ATP is stored in Ca^2+^ sensitive secretory vesicles, which are released upon activation through the BCR and TLR9 stimulation. Extracellular ATP is then hydrolyzed by membrane bound ectoenzymes to adenosine. The expression of one of those enzymes (ecto-5′-nucleotidases: 5′NT or CD73), as well as consequent adenosine production, correlates with naïve and IgM memory B cell propensity to undergo isotype class switch and differentiate into IgG and IgA secreting plasma cells. Remarkably, a significant decrease in CD73 expression was observed in a cohort of patients with common variable immunodeficiency (CVID), a disease characterized by hypogammaglobulinemia, increased susceptibility to infection, and autoimmunity. This result confirms previous observation of reduced 5′-nucleotidase activity in patients with hypo- and agammaglobulinemia [60, 61].

## 4. Immune Phenotypes of Dysfunction in Elements of Mitochondria

Primary mitochondrial disorders are a heterogeneous group of multisystem disorders linked to dysfunction of the mitochondrion, an organelle critical to cellular metabolism. These disorders are diagnosed via a multifaceted approach involving clinical assessment, family history, genetic and biochemical testing [62–64]. Current clinical criteria for primary mitochondrial disorders [53–55] include dysfunction of the central nervous, neuromuscular, cardiovascular, hematologic, gastrointestinal, endocrine, renal, ophthalmologic, auditory, hepatic, and dermatologic systems. Notably, immune dysfunction is not currently included in clinical diagnostic criteria for primary mitochondrial disorders.

Although immune dysfunction is not included in the current diagnostic criteria for primary mitochondrial disorders, a handful of monogenic disorders are known to result in immune deficiency involving perturbation of components (such as proteins and RNAs), coded by nuclear deoxyribonucleic acid (nDNA) and functioning in the mitochondrion. These disorders are not typically recognized as primary mitochondrial disorders and affected individuals would not necessarily score as likely to be affected by Bernier criteria [62]. Hereby we summarize dysfunction of three mitochondrial components described in the context of primary immunodeficiency not traditionally classified as primary mitochondrial disorders: adenylate kinase 2 (*AK2*), RNA component of mitochondrial RNA processing, endoribonuclease (*RMRP*), and* TAZ* genes (Table 1).

Adenylate kinase 2 (*AK2*) mutations have been associated with sensorineural deafness and reticular dysgenesis, a severe combined immune deficiency (SCID) with near absence of bone marrow lymphoid and myeloid elements [65]. Recently, a hypomorphic* AK2* mutation, with reduced enzyme expression, was identified in a case of inflammatory variant of leaky SCID, termed Omenn syndrome [66]. In the latter report, decreased T, B, and natural killer (NK) cell subsets were reported in conjunction with a very high frequency of activated/memory T cells (CD45RA) and poor T cell proliferation to mitogen. Curiously, based on the reported data, these patients would score as “unlikely” affected with a primary mitochondrial disorder by Bernier criteria [62].

The RNA component of mitochondrial RNA processing endoribonuclease (RMRP) contributes to ribosomal assembly, telomere function, and cell cycle control. Pathogenic mutations in the* RMRP* gene result in Cartilage Hair Hypoplasia (CHH) syndrome characterized by dwarfism, predisposition to infections, and variable degree of immune deficiency with decreased emigrant T cells as well as poor proliferation and increased apoptosis of activated peripheral T cells [67]. This presentation constitutes a “possible” primary mitochondrial disorder as per Bernier criteria [62].

Barth syndrome, secondary to mutation in the* TAZ* gene [Xq28], results in mitochondrial dysfunction (via cardiolipin deficiency), 3-methylglutaconic aciduria, cardioskeletal myopathy, and persistent or intermittent neutropenia, but with normal killing activity [68]. Based on reported data, these patients would score as “likely” affected with a primary mitochondrial disorder by Bernier criteria [62].

## 5. Susceptibility to Infection in Patients with Documented Mitochondrial Dysfunction

Conversely, while there are currently no primary immunodeficiencies also recognized as primary mitochondrial disorders, there is likewise limited data regarding immune dysfunction predisposition to infection in patients with diagnosed primary mitochondrial disorders. To date, remarkable rates of infection have been noted in cohorts specific to two primary mitochondrial diseases, mitochondrial neurogastrointestinal encephalomyopathy (MNGIE) [69] and carnitine palmitoyltransferase 1A deficiency [70], as well as in a single case report of a patient with multiple electron transport chain deficiencies [71], and a large cohort including patients with various primary mitochondrial disorders [72].

Currently available data on the rate of infections in patients with primary mitochondrial disorders is retrospective in design with variable control or baseline comparisons. In a retrospective clinical and genetic review of 92 patients with MNGIE—a well defined primary mitochondrial disorder presenting with gastrointestinal dysmotility, ptosis, ophthalmoplegia, hearing loss, and demyelinating peripheral neuropathy—7 (7.6%) were noted to have had recurrent infections during their disease course (follow-up time is not specified) [69]. Mutations of the* CPT1A *gene can lead to deficiency of CPT1A (the metabolic enzyme that controls mitochondrial fatty acid oxidation), an autosomal recessive metabolic disorder of long chain fatty acid oxidation characterized by severe episodes of hypoketotic hypoglycemia usually occurring after fasting or illness [73]. Recently, a study of the same hypomorphic* CPT1A* gene variant (Alaska variant) in a homozygous state in a large cohort (152 or 25% of 633 infants) of native Alaskan children demonstrated a statistically significant (to 95% confidence interval) association with increased frequency of lower respiratory tract infection (5.5 versus 3.7, *P* = 0.067) or acute otitis media (86% compared to 69%, 95% confidence interval, 1.4–8.9) compared to children who were heterozygous or noncarrier for this mutation. Of note, all medical care and clinical presentations included in the analysis occurred prior to the diagnosis of a* CPT1A *mutation and subgroup analysis was conducted to eliminate other potential confounders, including availability of healthcare facilities [70]. Interestingly, Reichenbach and colleagues have reported a case of an infant with electron transport chain complex II, III, and IV multiple enzyme deficiencies who presented with severe psychomotor retardation, axial hypotonia, and abnormal nociception. Immunological phenotyping revealed a decrease in all lymphocyte subsets, especially CD8 T cells, as well as hypogammaglobulinemia that required immunoglobulin substitutive therapy. T cell response to mitogens was normal. The only genetic finding was a heterozygous missense mutation of* POLG* of unclear significance [71].

We recently published a retrospective review [72] of the occurrence of infection and systemic inflammatory immune response syndrome, that is, SIRS [74], in a group of 97 patients with a clinical diagnosis of a primary mitochondrial disorder per published criteria [59] additionally supported by biochemical and/or genetic data. Among the 97 patients included in the analysis, newborn to 68 years old, primary mitochondrial disorders with supporting biochemical findings—primarily defects in oxidative phosphorylation demonstrated on muscle biopsy—constituted the majority (79 patients, 81%), and 34 (34%) patients had various supporting genetic findings (28 of whom carried a definitive molecular diagnosis). Patients experiencing recurrent urinary tract infections and no other infections were excluded due to the high likelihood of comorbid neurogenic bladder in mitochondrial disease. Bacteremia in the setting of an in-dwelling central venous line was deemed significant only if it occurred at a rate exceeding that described for pediatric patients with central venous lines (irrespective of underlying diagnosis) in a prospective surveillance trial [75]; pneumonia in the setting of respiratory insufficiency was only considered significant if the rate of pneumonia exceeded that reported in patients with nonmitochondrial neuromuscular disorders with similar ventilatory support and gastrostomy tube status [76]. Forty patients (42%) experienced serious or recurrent infections: 20 (21%) with bacterial, 7 (7.2%) with bacterial and fungal, 7 (7.4%) with bacterial and viral, and 6 (6.2%) with bacterial, fungal, and viral infections. Common pathogens included* Staphlococcus aureus *(15 patients affected),* Candida albicans *(8)*, Clostridium difficile* (6)*, Enterococcus *(5)*, Escherichia coli, Pseudomonas aeruginosa* (5), and respiratory syncytial virus (5). Twelve patients (13%) experienced 1 episode of sepsis or a more severe SIRS categorization. Three patients, aged 3 months, 2 years, and 3 years, died of severe and/or refractory septic shock. Twenty-seven of the 40 patients with serious or recurrent infection (68%) received laboratory immune testing. Nine (9% of the total cohort, 33% of patients with testing) had laboratory documented immunodeficiencies, including hypogammaglobulinemia (5 patients), transient hypogammaglobulinemia (1), and low IgA (1 patient), low vaccine titers (3), low avidity of anti-pneumococcal antibodies (1), low memory B cells (2), and transiently low T cells [72].

## 6. Conclusion

In summary, mitochondria perform essential roles in all eukaryotic organ systems, including both universally required and tissue-specific functions. Current laboratory studies and clinical observations are expanding our understanding of mitochondrial function in both the innate and adaptive immune systems, as well as documenting immune dysfunction in disorders affecting the mitochondrion. Further investigation—both bench and clinical—is required to further our understanding of the role of this critical organelle in immunity.

As prolonged infection can cause regression in milestones in many mitochondrial patients, an infection-free period not only improves quality of life but also enables mitochondrial patients to maximize their cognitive and physical development. On the other hand, patients with primary immunodeficiency, chronic infections, or inflammatory disease, may benefit from detailed evaluation of mitochondrial function and appropriate metabolic support to improve immune dysfunction.

## Figures and Tables

**Table 1:** Primary mitochondrial disorders are recognized as a group of multisystem disorders with various features and supporting laboratory findings [62]. Genetic or metabolic diagnoses—when identifiable—arise from perturbations of gene products localizing to the mitochondrion that may be nuclear or mitochondrially encoded [9] and are not necessary for a clinical diagnosis. The immunodeficiencies above have not typically been described as primary mitochondrial disorders but are linked to genetic defects of genes localizing to the mitochondrion. While published cases of Barth syndrome and Cartilage Hair Hypoplasia would be scored as “possible” or “likely affected” based on criteria by Bernier and colleagues, published cases of Omenn syndrome score as “unlikely affected” by a primary mitochondrial disorder.

Syndrome	Gene	Phenotype/immunologic phenotype	Bernier criteria classification
Barth syndrome	Tafazzin (*TAZ*)	3-Methylglutaconic aciduria, cardioskeletal myopathies/neutropenia	*Likely affected *

Omenn syndrome	*Adenylate kinase* (*AK*) *2*	Inflammatory variant of leaky severe combined immunodeficiency (L-SCID)	*Unlikely affected *

Cartilage Hair Hypoplasia	*Mitochondrial RNA processing endoribonuclease* (*RMRP*)	Dwarfism/predisposition to infections, variable immune deficiency with T cell dysfunction	*Possible *
